# Antenatal corticosteroids and perinatal outcome in late fetal growth restriction: analysis of prospective cohort

**DOI:** 10.1002/uog.26127

**Published:** 2023-02-01

**Authors:** A. Familiari, R. Napolitano, G. H. A. Visser, C. Lees, H. Wolf, F. Prefumo, the TRUFFLE‐2 feasibility study investigators, B. Arabin, A. Berger, E. Bergman, A. Bhide, C. M. Bilardo, A. C. Breeze, J. Brodszki, P. Calda, E. Cesari, I. Cetin, J. B. Derks, C. Ebbing, E. Ferrazzi, T. Frusca, W. Ganzevoort, S. J. Gordijn, W. Gyselaers, K. Hecher, P. Klaritsch, L. Krofta, P. Lindgren, S. M. Lobmaier, N. Marlow, G. M. Maruotti, F. Mecacci, K. Myklestad, B. Mylrea‐Foley, L. Raio, J. Richter, R. K. Sande, T. Stampalija, J. Thornton, H. Valensise, L. Wee

**Affiliations:** ^1^ Department of Woman and Child Health and Public Health Fondazione Policlinico Universitario A. Gemelli IRCCS Rome Italy; ^2^ Elizabeth Garrett Anderson Institute for Women's Health University College London London UK; ^3^ Fetal Medicine Unit University College London Hospitals NHS Foundation Trust London UK; ^4^ Department of Obstetrics University Medical Center Utrecht The Netherlands; ^5^ Centre for Fetal Care, Department of Obstetrics and Gynaecology Queen Charlotte's and Chelsea Hospital, Imperial College London London UK; ^6^ Department of Obstetrics and Gynecology Amsterdam University Medical Center (Location AMC), University of Amsterdam Amsterdam The Netherlands; ^7^ Obstetrics and Gynecology Unit IRCCS Istituto Giannina Gaslini Genoa Italy

**Keywords:** antenatal corticosteroids, fetal growth restriction, fetal lung maturation, late preterm

## Abstract

**Objective:**

To evaluate the role of antenatal administration of corticosteroids for fetal lung maturation on the short‐term perinatal outcome of pregnancy complicated by late fetal growth restriction (FGR).

**Methods:**

This cohort study was a secondary analysis of a multicenter prospective observational study, the TRUFFLE‐2 feasibility study, conducted between 2017 and 2018 in 33 European perinatal centers. The study included women with a singleton pregnancy from 32 + 0 to 36 + 6 weeks of gestation with a fetus considered at risk for FGR, defined as estimated fetal weight (EFW) and/or fetal abdominal circumference < 10^th^ percentile, or umbilicocerebral ratio (UCR) ≥ 95^th^ percentile or a drop of more than 40 percentile points in abdominal circumference measurement from the 20‐week scan. For the purposes of the current study, we identified women who received a single course of steroids to improve fetal lung maturation before delivery. Each exposed pregnancy was matched with one that did not receive antenatal corticosteroids (ACS) (control), based on gestational age at delivery and birth weight. The primary adverse outcome was a composite of abnormal condition at birth, major neonatal morbidity or perinatal death.

**Results:**

A total of 86 pregnancies that received ACS were matched to 86 controls. The two groups were similar with respect to gestational age (33.1 *vs* 33.3 weeks), EFW (1673 *vs* 1634 g) and UCR (0.68 *vs* 0.62) at inclusion, and gestational age at delivery (35.5 *vs* 35.9 weeks) and birth weight (1925 *vs* 1948 g). No significant differences were observed between the exposed and non‐exposed groups in the incidence of composite adverse outcome (28% *vs* 24%; *P* = 0.73) or any of its elements.

**Conclusion:**

The present data do not show a beneficial effect of steroids on short‐term outcome of fetuses with late FGR. © 2023 The Authors. *Ultrasound in Obstetrics & Gynecology* published by John Wiley & Sons Ltd on behalf of International Society of Ultrasound in Obstetrics and Gynecology.

**CONTRIBUTION**

*What are the novel findings of this work?*

This study showed no benefit of antenatal corticosteroids (ACS) for fetal lung maturation on short‐term perinatal outcome in pregnancies complicated by fetal growth restriction (FGR) after 32 weeks' gestation compared with matched pregnancies that did not receive ACS.

*What are the clinical implications of this work?*

This work supports the lack of evidence that ACS should be recommended routinely for late preterm FGR. To provide the best management for these pregnancies, it may be beneficial to identify if there is a subgroup of FGR that can benefit from ACS in order to maximize the potential benefits while minimizing risks.

## INTRODUCTION

Pregnancies affected by fetal growth restriction (FGR) are at increased risk of adverse obstetric outcome, particularly iatrogenic preterm delivery. Thus, administration of antenatal corticosteroids (ACS) to accelerate fetal lung maturation is the standard of care in order to reduce perinatal morbidity and mortality in these cases^1^. However, available studies focusing on FGR fetuses have not been able to confirm that respiratory distress syndrome (RDS) is reduced in FGR newborns after administration of ACS^2^, ^3^, ^4^, ^5^. While it is well‐established that appropriately grown fetuses at risk of preterm birth should be given a single course of ACS because the benefits exceed the risks^1^, it is still uncertain whether antenatal steroid exposure is beneficial, neutral or even detrimental in FGR.

Early studies demonstrated that lung growth and surfactant production are accelerated in FGR fetuses in the absence of antenatal glucocorticoid treatment^6^, probably as a result of the elevated plasma cortisol levels present in these cases^7^. Moreover, FGR fetuses have greater exposure to maternal steroids through the downregulation of placental 11‐beta‐hydroxysteroid dehydrogenase type II (11‐bHSD‐II), the enzyme that normally prevents maternal cortisol from crossing the placenta^8^. Research findings are conflicting, with some studies reporting a similar incidence of RDS in FGR and in non‐FGR babies^5^, ^9^, ^10^, and others an increased risk of RDS in FGR newborns^11^. Currently, it is still under debate whether ACS are^12^ or are not^13^ associated with a beneficial reduction in complications in FGR newborns. Torrance *et al*.^14^ suggested that ACS treatment does not affect mortality or morbidity in FGR fetuses. In FGR animal models, antenatal steroids have been shown to reduce fetal brain growth, alter cerebral blood flow and cause brain damage^15^, ^16^, raising the question as to whether antenatal administration of steroids in late FGR fetuses could be detrimental.

Given these premises, we aimed to investigate the role of ACS on perinatal outcome in pregnancy complicated by late FGR.

## METHODS

This was a secondary analysis of the TRUFFLE‐2 feasibility study, a multicenter prospective observational cohort study conducted between 1 April 2017 and 1 July 2018 in 33 European perinatal centers^17^. Briefly, women were eligible if they had a singleton pregnancy from 32 + 0 to 36 + 6 weeks of gestation with a fetus considered at risk for FGR, defined as estimated fetal weight (EFW) and/or fetal abdominal circumference (AC) < 10^th^ percentile, or umbilicocerebral ratio (UCR) ≥ 95^th^ percentile, or a drop of more than 40 percentile points in AC measurement from the 20‐week scan. The references for EFW, AC and Doppler parameters were based on local charts. In order to be eligible, the fetus had to have positive umbilical artery end‐diastolic flow and a normal computerized cardiotocogram with a short‐term variability of > 3.0 ms. Gestational age was calculated based on the last menstrual period (if certain) and/or ultrasound assessment before 22 weeks of gestation. Women were ineligible for inclusion if there was planned or impending delivery based either on maternal obstetric complications, uterine contractions or rupture of membranes, or if their fetus had known or suspected structural or chromosomal abnormality. Birth‐weight *Z*‐scores were calculated using the Hadlock fetal growth chart^18^. Data were collected on a secure cloud‐based electronic data capture platform (Castor EDC, Amsterdam, The Netherlands). The database carried no personal identifiers. Participants and their infants could be identified only using unique study identifiers that were stored in their recruiting center.

For the purposes of the current study, we identified women who received steroids to improve fetal lung maturation before delivery (exposed), according to each participating institution's local policy or clinician advice. All units considered as a single course of steroids when a total dosage of 24 mg of betamethasone or dexamethasone was administered intramuscularly or intravenously in multiple doses over a period of 48 h. Each exposed pregnancy was matched with one that did not receive ACS (non‐exposed), based on gestational age at delivery ± 10 days and birth weight ± 150 g.

The primary adverse outcome was a composite of abnormal condition at birth, major neonatal morbidity or perinatal death. Abnormal condition at birth was defined as at least one of the following: 5‐min Apgar score < 7, umbilical artery pH < 7.0 or umbilical vein pH < 7.1, resuscitation with intubation, chest compressions or need for medication. Major neonatal morbidity was defined as at least one of the following: neurological abnormality (intracranial hemorrhage Grade 3 or 4, periventricular leukomalacia Grade 2 or 3, encephalopathy or seizures necessitating antiepileptic drug treatment); cardiovascular abnormality (hypotensive treatment, ductus arteriosus treatment or disseminated coagulopathy); respiratory morbidity (respiratory support for more than 1 week, mechanical ventilation, meconium aspiration or persistent pulmonary hypertension); or sepsis (clinical sepsis with positive blood culture, necrotizing enterocolitis (Bell's Stage 2 or greater) or meningitis).

Categorical data are presented as *n* (%) and continuous data as median (interquartile range). Background data and perinatal outcome were compared between the exposed and non‐exposed groups using Kruskal–Wallis test or Fisher's exact test, as appropriate. Statistical analysis was performed using SPSS software (version 25; IBM Corp., New York, NY, USA).

## RESULTS

**Figure 1:**
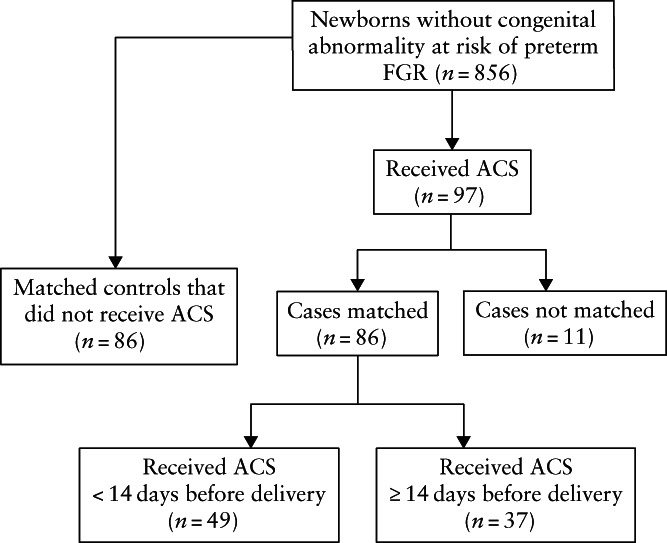
Flowchart showing inclusion in study cohort of pregnancies with fetal growth restriction (FGR) after 32 weeks that received antenatal corticosteroids (ACS) and pregnancies that did not (controls), matched for gestational age at delivery and birth weight.

Complete delivery and outcome data were available for 856 newborns without major congenital abnormalities. Of these, 97 pregnancies received a single course of steroids, comprising 83 (86%) that received betamethasone and 14 (14%) that received dexamethasone, which was given within 2 weeks before delivery in 57 (59%). Repeat courses were not administered. A total of 86 (exposed) pregnancies were matched to 86 non‐exposed controls (Figure 1). There were 11 pregnancies that received steroids but could not be matched to a non‐exposed one because gestational age at delivery and birth weight were too low.

Demographic, obstetric and fetal Doppler velocimetry characteristics of the pregnancies included in the cohort are shown in Table 1. The exposed and non‐exposed groups were similar with respect to gestational age at inclusion, EFW and UCR, gestational age at delivery and birth weight. The overall median gestational age at inclusion was 33 weeks and EFW was 1673 g. There was no statistically significant difference in the incidence of composite adverse outcome between the exposed and non‐exposed groups (28% *vs* 24%; *P* = 0.73). Women who received ACS within 14 days before delivery, compared to those with an interval ≥ 14 days, had significantly higher gestational age at steroid administration (34.4 *vs* 31.9 weeks; *P* < 0.01), lower gestational age at delivery (35.3 *vs* 36.9 weeks; *P* = 0.01) and higher rate of composite adverse outcome (39% *vs* 14%; *P* = 0.01) (Table 1). The 11 exposed women who could not be matched had a significantly lower EFW and higher UCR at inclusion, and lower birth weight and gestational age at delivery compared with the matched ACS group. This explains the inability to match these pregnancies.

**Table 1:** Demographic, obstetric and Doppler variables in 86 pregnancies with late fetal growth restriction that received antenatal corticosteroids (ACS), overall and according to treatment‐to‐delivery interval, 86 matched pregnancies that did not receive ACS (controls), 11 pregnancies that received ACS but could not be matched and 759 pregnancies that did not receive ACS

	ACS									
Variable	Interval < 14 days (*n* = 49)	Interval ≥ 14 days (*n* = 37)	*P* *	All (*n* = 86)	Controls (*n* = 86)	*P* †	Unmatched ACS (*n* = 11)	*P* ‡	No ACS (*n* = 759)§	*P* ¶
Maternal age (years)	33 (29.5–37.0)	32 (28.5–34.0)	0.26	32 (29.0–36.0)	32 (28.0–36.0)	0.35	33 (28.0–35.0)	0.73	31 (28.0–35.0)	0.03
Nulliparous	32 (65.3)	24 (64.9)	1.00	56 (65.1)	51 (59.3)	0.53	9 (81.8)	0.39	459 (60.5)	0.23
Smoker	1 (2.0)	4 (10.8)	0.21	5 (5.8)	9 (10.5)	0.40	0 (0)	0.92	63 (8.3)	0.65
BMI (kg/m^2^)	24.1 (21.0–29.0)	24.2 (19.9–27.1)	0.26	24.2 (20.8–27.6)	22.5 (20.4–25.4)	0.25	24.2 (22.4–26.9)	0.38	22.3 (20.2–25.4)	0.01
PIH	20 (40.8)	14 (37.8)	0.82	34 (39.5)	34 (39.5)	1.00	7 (63.6)	0.13	78 (10.3)	< 0.01
GA at inclusion (weeks)**	33.4 (32.4–34.7)	33.0 (32.4–34.5)	0.52	33.1 (32.4–34.5)	33.3 (32.4–34.6)	0.97	32.3 (32.1–32.7)	0.02	34.1 (32.9–35.6)	< 0.01
EFW at inclusion (g)**	1684 (1439–1922)	1584 (1377–1899)	0.45	1673 (1392–1905)	1634 (1462–1892)	0.78	1331 (1252–1405)	< 0.01	1920 (1668–2169)	< 0.01
UCR at inclusion**	0.69 (0.53–0.83)	0.66 (0.47–0.84)	0.73	0.68 (0.51–0.83)	0.62 (0.51–0.75)	0.22	1.02 (0.75–1.95)	< 0.01	0.55 (0.47–0.66)	< 0.01
GA at ACS (weeks)	34.4 (33.7–35.8)	31.9 (30.2–33.2)	< 0.01	33.8 (32.2–35.3)	—	—	32.1 (31.3–32.3)	< 0.01	—	—
Duration of ACS (days)	4 (3–7)	28 (20–45)	< 0.01	8 (3–25)	—	—	10 (2–15)	0.31	—	—
GA at delivery (weeks)	35.3 (34.2–36.4)	36.9 (34.8–37.8)	0.01	35.5 (34.4–37.0)	35.9 (34.9–37.0)	0.34	33.0 (32.3–33.9)	< 0.01	38.3 (37.1–39.3)	< 0.01
Birth weight (g)	1880 (1720–2090)	2020 (1760–2250)	0.16	1925 (1714–2200)	1948 (1718–2170)	0.91	1220 (1165–1300)	< 0.01	2544 (2250–2820)	< 0.01
Birth‐weight *Z*‐score	−2.3 (−2.7 to −1.7)	−2.6 (−3.0 to −2.0)	0.27	−2.5 (−2.9 to −1.8)	−2.6 (−3.0 to −2.0)	0.46	−3.5 (−4.1 to −3.3)	< 0.01	−1.9 (−2.4 to −1.4)	< 0.01
Composite adverse outcome††	19 (38.8)	5 (13.5)	0.01	24 (27.9)	21 (24.4)	0.73	8 (72.7)	0.01	61 (8.0)	< 0.01

Data are given as median (interquartile range) or *n* (%).

Comparisons were performed using Fisher's exact test or Kruskal–Wallis test between:

* matched ACS pregnancies with treatment‐to‐delivery interval < 14 days (*n* = 49) *vs* ≥ 14 days (*n* = 37);

† total matched ACS pregnancies (*n* = 86) *vs* controls (*n* = 86);

‡ total matched ACS pregnancies (*n* = 86) *vs* not matched ACS pregnancies (*n* = 11);

¶ pregnancies that did not receive ACS (*n* = 759) *vs* all that received treatment (*n* = 97).

§ Group includes controls.

** At time of inclusion in TRUFFLE‐2 feasibility study^17^.

†† Defined as abnormal condition at birth, major neonatal morbidity or perinatal death.

BMI, body mass index; EFW, estimated fetal weight; GA, gestational age; PIH, pregnancy‐induced hypertension; UCR, umbilicocerebral ratio.

Women who received ACS, compared with those who did not, had higher obstetric risk (higher age, body mass index and rate of hypertensive morbidity), were included at an earlier gestational age, had lower EFW and higher UCR, and delivered at an earlier gestational age a neonate with lower birth weight, associated with a higher rate of composite adverse outcome (Table 1).

**Figure 2:**
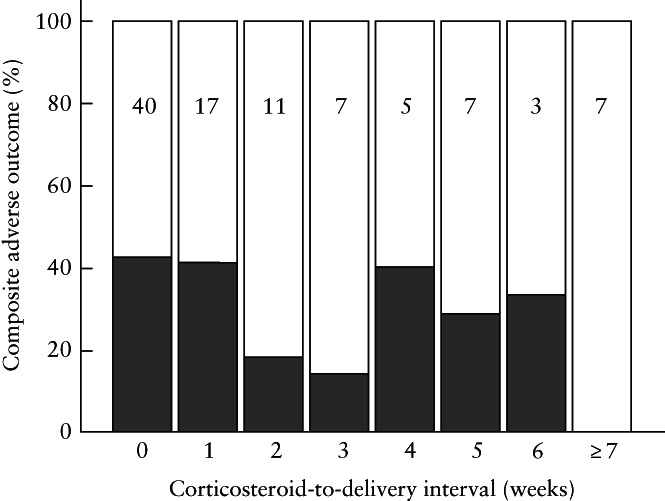
Incidence of composite adverse outcome in 97 infants with late fetal growth restriction that received antenatal corticosteroids, according to interval between corticosteroid administration and delivery. Total number of infants in each week is shown in bars.

Delivery details and perinatal outcome of the exposed and non‐exposed groups are further specified in Table 2. The median gestational age at delivery was 35 weeks in both groups. Pregnancies that received ACS were delivered more frequently by prelabor Cesarean section. No significant differences were observed between the exposed and non‐exposed groups in the rate of composite adverse outcome or any of its elements. When comparing pregnancies that received ACS within 14 days before delivery with their matched non‐exposed pregnancies, there was a higher rate of major neonatal morbidity (37% *vs* 16%; *P* = 0.04) and respiratory morbidity (31% *vs* 12%; *P* = 0.05) in the ACS group, whereas all other parameters were similar between the two groups (Table S1). A similar comparison between women who received corticosteroids ≥ 14 days before delivery *vs* their matched non‐exposed pregnancies showed no statistically significant differences in perinatal and outcome parameters (Table S2). Figure 2 shows the percentage of adverse composite outcome in all infants who received ACS (*n* = 97), according to the interval between corticosteroid administration and delivery.

**Table 2:** Delivery characteristics and perinatal outcome of 86 pregnancies with late fetal growth restriction (FGR) that received antenatal corticosteroids (ACS) and 86 matched pregnancies that did not receive ACS

	ACS	No ACS	
Parameter	(*n* = 86)	(*n* = 86)	*P* *
Mode of delivery			0.01
Spontaneous labor			
Vaginal delivery	4 (4.7)	14 (16.3)	
CS	11 (12.8)	3 (3.5)	
Induction			
Vaginal delivery	12 (14.0)	17 (19.8)	
CS	4 (4.7)	11 (12.8)	
Prelabor CS	55 (64.0)	41 (47.7)	
Indication for prelabor CS			0.40
Fetal†	30/55 (54.5)	17/41 (41.5)	
Maternal	11/55 (20.0)	9/41 (22.0)	
Other	14/55 (25.5)	15/41 (36.6)	
Perinatal outcome			
GA at delivery (weeks)	35.5 (34.4–37.0)	35.9 (34.9–37.0)	0.34
Birth weight (g)	1925 (1714–2200)	1948 (1718–2170)	0.91
Birth‐weight *Z*‐score	−2.5 (−2.9 to −1.8)	−2.6 (−3.0 to −2.0)	0.46
Male sex	39 (45.3)	38 (44.2)	1.00
Abnormal condition atbirth	6 (7.0)	6 (7.0)	1.00
Major neonatalmorbidity‡	23 (26.7)	18 (20.9)	0.47
Cerebral morbidity	0 (0)	0 (0)	—
Cardiovascularmorbidity	2 (2.3)	4 (4.7)	0.68
Infection/sepsis	4 (4.7)	3 (3.5)	1.00
Respiratory morbidity	20 (23.3)	11 (12.8)	0.11
Respiratory support			
Before first week	16 (18.6)	8 (9.3)	0.12
After first week	0 (0)	0 (0)	—
Mechanicalventilation	2 (2.3)	0 (0)	0.50
RDS	7 (8.1)	3 (3.5)	0.33
Other respiratorymorbidity	1 (1.2)	1 (1.2)	1.00
Composite adverseoutcome§	24 (27.9)	21 (24.4)	0.73

Data are given as *n* (%), *n*/*N* (%) or median (interquartile range).

* Fisher's exact test or Kruskal–Wallis test.

† Computerized cardiotocogram, Doppler, FGR.

‡ Some cases had multiple diagnoses.

§ Defined as abnormal condition at birth, major neonatal morbidity or perinatal death.

CS, Cesarean section; GA, gestational age; RDS, respiratory distress syndrome.

## DISCUSSION

This study showed no benefit of administration of ACS for fetal lung maturation or other neonatal morbidity in pregnancies complicated by FGR after 32 weeks of gestation. Composite adverse outcome and other delivery and neonatal outcomes were similar between exposed and non‐exposed pregnancies. Only 11% of the study population received corticosteroids. The decision to administer corticosteroids was left to the individual clinician and was apparently guided by the perception of increased perinatal risk. Women who received corticosteroids had a lower gestational age at inclusion, lower EFW and higher UCR compared to those who did not.

The present data do not show a beneficial effect of ACS on short‐term outcome in a prospectively selected and appropriately FGR phenotyped cohort. The small sample size and the fact that no matched control could be found for the 11 women with the highest risk represent limitations of the study given the possibility that a type‐II error may still be present, and do not allow definitive conclusions to be drawn regarding the benefit or disadvantages of steroids in FGR^19^. An adequately powered study to explore significantly and clinically meaningful differences in reducing composite adverse outcome from 21% to 10% would require 225 women per intervention arm (90% power, alpha 0.05 and beta 0.1). However, meaningful conclusions from this analysis can be evaluated to generate the research hypothesis. Another limitation of the study is that evaluation of the effect of steroids on perinatal outcome was not the aim of the TRUFFLE‐2 feasibility study, the latter being designed to explore the best predictors of outcome in order to investigate in a randomized trial the optimal timing of delivery in late FGR^20^. Additionally, receiving corticosteroids < 14 days before delivery may be associated with a higher obstetric risk and bias results. In this regard, it should be noted that a non‐statistically significant, but possibly clinically relevant, difference in the incidence of adverse outcome was observed between the exposed and non‐exposed groups both for a treatment‐to‐delivery interval < 14 days (39% *vs* 20%) and ≥ 14 days (14% *vs* 30%). However, as shown in Figure 2, the relationship between treatment‐to‐delivery interval and outcome seems to be more complex, and our limited sample size does not allow further investigation.

The main strength of this study is that the effects of steroids on perinatal outcome of late FGR were evaluated in a selected population of late FGR fetuses followed up prospectively until delivery. By matching on birth weight and gestational age at delivery, exposed and non‐exposed pregnancies were similar concerning the two most relevant predictors of adverse perinatal outcome.

In the first trial on the effects of steroids in cases of preterm birth, a non‐significantly higher fetal mortality was reported in cases of severe maternal hypertension and FGR^21^ and consequently FGR was excluded from subsequent trials. Therefore, obstetricians must base clinical practice on observational studies only. The present data on short‐term outcome are in line with several papers showing no significant beneficial effect of steroid administration in the context of FGR^5^, ^13^, ^14^, ^19^. Only one of these studies targeted late preterm FGR^19^, similar to ours, and did not observe a decrease in respiratory morbidity following ACS administration. However, other studies on early preterm neonates and those born with FGR observed a reduction in cerebral hemorrhage^22^, a reduced risk of RDS, intraventricular hemorrhage and perinatal death^12^ or an increase in survival without disability or impairment at 2 years of age^23^ following ACS administration. The reported inconsistencies regarding the effect of steroids on neonatal outcomes in normally grown compared to FGR babies may be due to differences in gestational age or duration of exposure at ACS administration, or effects of glucocorticoids on the development of organ systems.

The role of steroids in appropriately grown late preterm infants has been studied extensively and benefits have been demonstrated^24^. However, the possible effect of ACS on the subgroup of fetuses affected by FGR is still under debate. It has been postulated that FGR itself may lead to enhanced fetal lung maturation through different mechanisms: chronic intrauterine stress seems to stimulate production of cortisol by the fetal adrenal gland, and the downregulation of placental 11‐bHSD‐II increases exposure to maternal steroids in these fetuses^7^, ^14^. Assuming that FGR fetuses are exposed to increased levels of cortisol, we can hypothesize that even a single course of ACS acts like a repeat dose, thus explaining why exogenous administration of glucocorticoids may have no additional benefit in FGR fetuses or possibly be detrimental both in the short and long term^25^, ^26^.

Recent evidence suggests that the use of glucocorticoids in the perinatal period could be associated with adverse effect on neurodevelopmental outcomes^15^, ^16^, ^25^, ^26^, ^27^. It has also been demonstrated that repeated administration of steroids to mothers at risk of preterm birth can adversely affect fetal growth, induce hypertension and reduce brain growth with delayed myelination^5^, ^15^. A retrospective study of 247 pregnancies with FGR or small‐for‐gestational‐age fetuses found that ACS in the late preterm period did not decrease significantly the need for respiratory support in newborns, while the rate of neonatal hypoglycemia increased significantly after ACS exposure^19^. However, following the results of the Antenatal Late Preterm Steroids (ALPS) trial^27^, the American College of Obstetricians and Gynecologists and the Society of Maternal–Fetal Medicine recommended steroid administration for late preterm pregnancies without prior exposure to ACS and at risk of delivery within the next 7 days^28^. FGR is a risk factor for iatrogenic preterm birth, making growth‐restricted infants more likely to be exposed to both early and late preterm steroids, with possibly no or limited benefit and tangible risks.

In conclusion, our findings highlight the need for studies focusing on the effect of ACS in late preterm FGR. In order to provide the best prenatal management for these pregnancies, it may be necessary to identify whether there is a subgroup of FGR that can benefit from ACS, and if so, to evaluate the best timing of this intervention in order to maximize the potential benefits while minimizing risks. At present, we believe that there is insufficient evidence to recommend ACS to be given routinely in the context of late preterm FGR.

## Supporting information


**Table S1** Delivery characteristics and perinatal outcome of 49 pregnancies that received antenatal corticosteroids (ACS) < 14 days before delivery and 49 matched pregnancies that did not

**Table S2** Delivery characteristics and perinatal outcome of 37 pregnancies that received antenatal corticosteroids (ACS) ≥ 14 days before delivery and 37 matched pregnancies that did not

## Data Availability

The data that support the findings of this study are available from the corresponding author upon reasonable request.
