# Supplementary material for: Antenatal corticosteroids and perinatal outcome in late fetal growth restriction: analysis of prospective cohort
Source: Ultrasound Obstet Gynecol. 2023 Feb 1;61(2):191–7. doi: 10.1002/uog.26127 (PMC10108243; doi:10.1002/uog.26127)
Supplement: Supplementary file 1 — Table S1 Delivery characteristics and perinatal outcome of 49 pregnancies that received antenatal corticosteroids (ACS) < 14 days before delivery and 49 matched pregnancies that did not [file UOG-61-191-s001.docx]

**Table S1.** Delivery characteristics and perinatal outcome of 49 pregnancies that received antenatal corticosteroids (ACS) < 14 days before delivery and 49 matched pregnancies that did not

|  | ACS (n=49) | No ACS (n=49) | P# |
| --- | --- | --- | --- |
| Inclusion |  |  |  |
| Gestational age | 33.4 (32.4-34.7) | 33.3 (32.5-34.8) | 0.84 |
| EFW | 1684 (1439-1923) | 1636 (1462-1867) | 0.52 |
| UCR | 0.69 (0.53-0.83) | 0.66 (0.51-0.86) | 0.91 |
| Gestational age at corticosteroids | 34.4 (33.7-35.8) |  |  |
|  |  |  |  |
| Perinatal outcome |  |  |  |
| Gestational age at delivery (weeks) | 35.3 (34.2-36.4) | 35.6 (34.7-36.4) | 0.29 |
| Birth weight (g) | 1880 (1720-2090) | 1900(1710-2098) | 0.90 |
| Male sex | 28 (57) | 25 (51) | 0.69 |
| Abnormal condition at birth | 4 (8) | 3 (6) | 1.00 |
| Major neonatal morbidity* | 18 (37) | 8 (16) | **0.04** |
| Cerebral morbidity | 0 (0) | 0 (0) | --- |
| Cardiovascular morb. | 2 (4) | 0 (0) | 0.50 |
| Infection/sepsis | 4 (8) | 2 (4) | 0.68 |
| Respiratory morbidity | 15 (31) | 6 (12) | **0.05** |
| Resp. support <1^st^ wk | 12 (25) | 5 (10) | 0.11 |
| Resp. support after 1^st^ wk | 0 | 0 | --- |
| Mechanical ventilation | 1 (2) | 0 (0) | 1.00 |
| RDS | 6 (12) | 1 (2) | 0.11 |
| Other resp. morbidity | 0 | 0 | --- |
| Composite adverse outcome | 19 (39) | 10 (20) | 0.07 |

Data are given as median (interquartile range) or n (%).

CS: Cesarean section

* Multiple diagnoses possible

# Fisher Exact Test or Kruskal-Wallis Test
