# Supplementary material for: Antenatal corticosteroids and perinatal outcome in late fetal growth restriction: analysis of prospective cohort
Source: Ultrasound Obstet Gynecol. 2023 Feb 1;61(2):191–7. doi: 10.1002/uog.26127 (PMC10108243; doi:10.1002/uog.26127)
Supplement: Supplementary file 2 — Table S2 Delivery characteristics and perinatal outcome of 37 pregnancies that received antenatal corticosteroids (ACS) ≥ 14 days before delivery and 37 matched pregnancies that did not [file UOG-61-191-s002.docx]

**Table S2.** Delivery characteristics and perinatal outcome of 37 pregnancies that received antenatal corticosteroids (ACS) ≥ 14 days before delivery and 37 matched pregnancies that did not

|  | ACS (n=37) | No ACS (n=37) | P# |
| --- | --- | --- | --- |
| Inclusion |  |  |  |
| Gestational age | 33.0 (32.4-34.5) | 33.1 (32.4-34.5) | 0.83 |
| EFW | 1584 (1377-1900) | 1626 (1456-1917) | 0.77 |
| UCR | 0.66 (0.47-0.84) | 0.61 (0.50-0.67) | 0.12 |
| Gestational age at corticosteroids | 31.9 (30.2-33.2) | --- | --- |
|  |  |  |  |
| Perinatal outcome |  |  |  |
| Gestational age at delivery (weeks) | 36.9 (34.8-37.8) | 36.7 (35.2-37.7) | 0.71 |
| Birth weight (g) | 2020 (1760-2250) | 2020 (1731-2288) | 0.93 |
| Male sex | 11 (30) | 13 (35) | 0.80 |
| Abnormal condition at birth | 2 (5) | 3 (8) | 1.00 |
| Major neonatal morbidity* | 5 (14) | 10 (27) | 0.25 |
| Cerebral morbidity | 0 (0) | 0 (0) | --- |
| Cardiovascular morbidity | 0 (0) | 4 (11) | 0.12 |
| Infection/sepsis | 0 (0) | 1 (3) | 1.00 |
| Respiratory morbidity | 5 (14) | 5 (14) | 1.00 |
| Resp. support <1^st^ wk | 4 (11) | 3 (8) | 1.00 |
| Resp. support after 1^st^ wk | 0 (0) | 0 (0) | --- |
| Mechanical ventilation | 1 (3) | 0 (0) | 1.00 |
| RDS | 1 (3) | 2 (5) | 1.00 |
| Other resp. morbidity | 1 (3) | 1 (3) | 1.00 |
| Composite adverse outcome | 5 (14) | 11 (30) | 0.16 |

Data are given as median (interquartile range) or n (%).

CS: Cesarean section

* Multiple diagnoses possible

# Fisher Exact Test or Kruskal-Wallis Test
